# Supplementary material for: RNA interference as a gene silencing tool to control Tuta absoluta in tomato (Solanum lycopersicum)
Source: PeerJ. 2016 Dec 15;4:e2673. doi: 10.7717/peerj.2673 (PMC5162399; doi:10.7717/peerj.2673)
Supplement: Figure S1 — For Vacuolar ATPase subunit-A: Aedes aegypti ( XP_001659520.1); Drosophila melanogaster ( NP_652004.2); Tribolium castaneum ( XP_976188.1); Manduca sexta (P31400.1); Bombyx mori ( NP_001091829.1); Tuta absoluta (KM591219). Arginine Kinase: Spodoptera litura (ADW94627.1); Helicoverpa armigera (ADD22718.1); Bombyx mori ( NP_001037402.1); Tribolium castaneum (EFA11419.1); Homalodisca vitripennis (AAT01074.1); Drosophila melanogaster (AAA68172.1); Tuta absoluta (KM591220). [file peerj-04-2673-s001.pdf]

Vascular ATPase subunit A

```
            391     400       410       420       430       440       450       460       470       480       490       500       510       520
            |--------+---------+---------+---------+---------+---------+---------+---------+---------+---------+---------+---------+---------|
     Aedes  RVKCLGNPEREGSVSIVGAVSPPGGDFSDPVTSATLGIVQVFWGLDKKLAQRKHFPSINWLISYSKYMRALDDFYDKNFQEFVPLRTKVKEILQEEEDLSEIVQLVGKASLAETDKITLEVAKLLKDDFL
Drosophila  RVKCLGNPEREGSVSIVGAVSPPGGDFSDPVTSATLGIVQVFWGLDKKLAQRKHFPSINWLISYSKYMRALDDFYDKNFPEFVPLRTKVKEILQEEEDLSEIVQLVGKASLAETDKITLEVAKLLKDDFL
 Tribolium  RVKCLGNPDREGSVSIVGAVSPPGGDFSDPVTSATLGIVQVFWGLDKKLAQRKHFPSINWLISYSKYTRALDDFYDKNFQEFVALRTKVKEILQEEEDLSEIVQLVGKASLAETDKITLEIAKLLKEDFL
   Manduca  RVKCLGNPDREGSVSIVGAVSPPGGDFSDPVTAATLGIVQVFWGLDKKLAQRKHFPSINWLISYSKYMRALDDFYEKNYPEFVPLRTKVKEILQEEEDLSEIVQLVGKASLAETDKITLEVAKLLKDDFL
    Bombyx  RVKCLGNPDREGSVSIVGAVSPPGGDFSDPVTAATLGIVQVFWGLDKKLAQRKHFPSINWLISYSKYMRALDDFYEKNYPEFVPLRTKVKEILQEEEDLSEIVQLVGKASLAETDKITLEVAKLLKDDFL
      Tuta                            LGPGILGSIFDGIQRPLKDISELTQSIHFPSINWLISYSKYMRALDDFYDKNYPEFVPLRTKVKEILQEEEDLSEIVQLVGKASLAETDKITLEVAKLLKDDFL
 Consensus  rvkclgnp.regsvsivgavsppggdfsdp!t.atlgi!Qvfwgldkkl aQrkHFPSINWLISYSKYmRALDDFY#KN%pEFVpLRTKVKEILQEEEDLSEIVQLVGKASLAETDKITLE!AKLLK#DFL

            521     530       540       550       560       570       580       590       600       610    617
            |--------+---------+---------+---------+---------+---------+---------+---------+---------+------|
     Aedes  QQNSYSAYDRFCPFYKTVGMLRNMIGFYDMARHAVETTAQSENKITWNVIRDSMGNILYQLSSMKFKDPVKDGEAKIKADFDQLYEDLQQAFRNLED
Drosophila  QQNSYSSYDRFCPFYKTVGMLRNIIDFYDMARHSVESTAQSENKITWNVIREAMGNIMYQLSSMKFKDPVKDGEAKIKADFEQLHEDLQQAFRNLED
 Tribolium  QQNSYSSYDRFCPFYKTVGMLKNMIGLYDMSRHAVESTAQSENKITWTVIRDSMSNILYQLSSMKFKDPVKDGEAKIKADFDQLYEDIQQAFRNLED
   Manduca  QQNSYSSYDRFCPFYKTVGMLKNIISFYDMSRHAVESTAQSDNKVTWNVIRDAMGNVLYQLSSMKFKDPVKDGEAKIKADFDQLLEDMSAAFRNLED
    Bombyx  QQNSYSSYDRFCPFYKTVGMLKNIITFYDMSRHAVESTAQSDNKVTWNVIRDAHGHVLYQLSSMKFKDPVKDGEPKIKADFDQLLEDMSAAFRNLED
      Tuta  QQNSYSAYDRFCPLYKQ
 Consensus  QQNSYSaYDRFCPfYKtvgml.n.i..ydm.rh.ve.taqs.nk.tw.vir.......yqlssmkfkdpvkdge.kikadf.ql.ed...afrnled
```

Arginine Kinase

```
             261     270       280       290       300       310       320       330       340       350       360       370       380       390
             |--------+---------+---------+---------+---------+---------+---------+---------+---------+---------+---------+---------+---------|
 Spodoptera  RVRCGRSMEGYPFNPCLTEAQYKEMEEKVASTLSGLEGELKGTFYPLTGMSKETQQQLIDDHFLFKEGDRFLQAANACRFWPSGRGIYHNENKTFLVWCNEEDHLRLISMQMGGDLKQVYKRLVTAVNDI
Helicoverpa  RVRCGRSMEGYPFNPCLTEAQYKEMEEKVSSTLSGLEGELKGTFYPLTGMSKETQQQLIDDHFLFKEGDRFLQAANACRFWPTGRGIYHNENKTFLVWCNEEDHLRLISMQMGGDLKQVYKRLVTAVNDI
     Bombyx  RVRCGRSLEGYPFNPCLTESQYKEMEDKVSGTLSSLEGELKGTFYPLTGMSKETQQQLIDDHFLFKEGDRFLQAANACRFWPTGRGIYHNENKTFLVWCNEEDHLRIISMQMGGDLQQVYKRLVSAVNEI
  Tribolium  RVRCGRSLEGYPFNPCLTEEQYKEMEQKVSSTLSGLEGELKGTFYPLTGMSKEVQQKLIDDHFLFKEGDRFLQAANACRFWPTGRGIFHNDAKTFLVWCNEEDHLRIISMQMGGDLGQVYRRLVTGVNDI
Homalodisca  RVRCGRSMEGYPFNPCLTEAQYKEMEEKVSSTLSGLEGELKGAFYPLTGMTKEVQQKLIDDHFLFKEGDRFLQAANACRFWPTGRGIYHNDNKTFLVWCNEEDHLRLISMQMGGDLGQVYRRLVTAVNDI
 Drosophila  RVRCGRSMQGYPFNPCLTEAQYKEMESKVSSTLSGLEGELKGKFYPLTGMEKAVQQQLIDDHFLFKEGDRFLQAANACRFWPSGRGIYHNDAKTFLVWCNEEDHLRIISMQQGGDLGQIYKRLVTAVNEI
       Tuta                   EAQYKEMEDKVSSTLSGLEGELKGTFYPLTGMSKETQQQLIDDHFLFKEGDRFLQAANACRFWPTGRGIYHNENKTFLVWCNEEDHL
  Consensus  rvrcgrs..gypfnpcltEaQYKEMEdKVSSTLSGLEGELKGtFYPLTGMsKetQQqLIDDHFLFKEGDRFLQAANACRFWPtGRGI%HN#nKTFLVWCNEEDHLr.ismq.ggdl.q.y.rlv..vn.i
```
