# Supplementary figures and images for: RNA interference as a gene silencing tool to control Tuta absoluta in tomato (Solanum lycopersicum)
Source: PeerJ. 2016 Dec 15;4:e2673. doi: 10.7717/peerj.2673 (PMC5162399; doi:10.7717/peerj.2673)

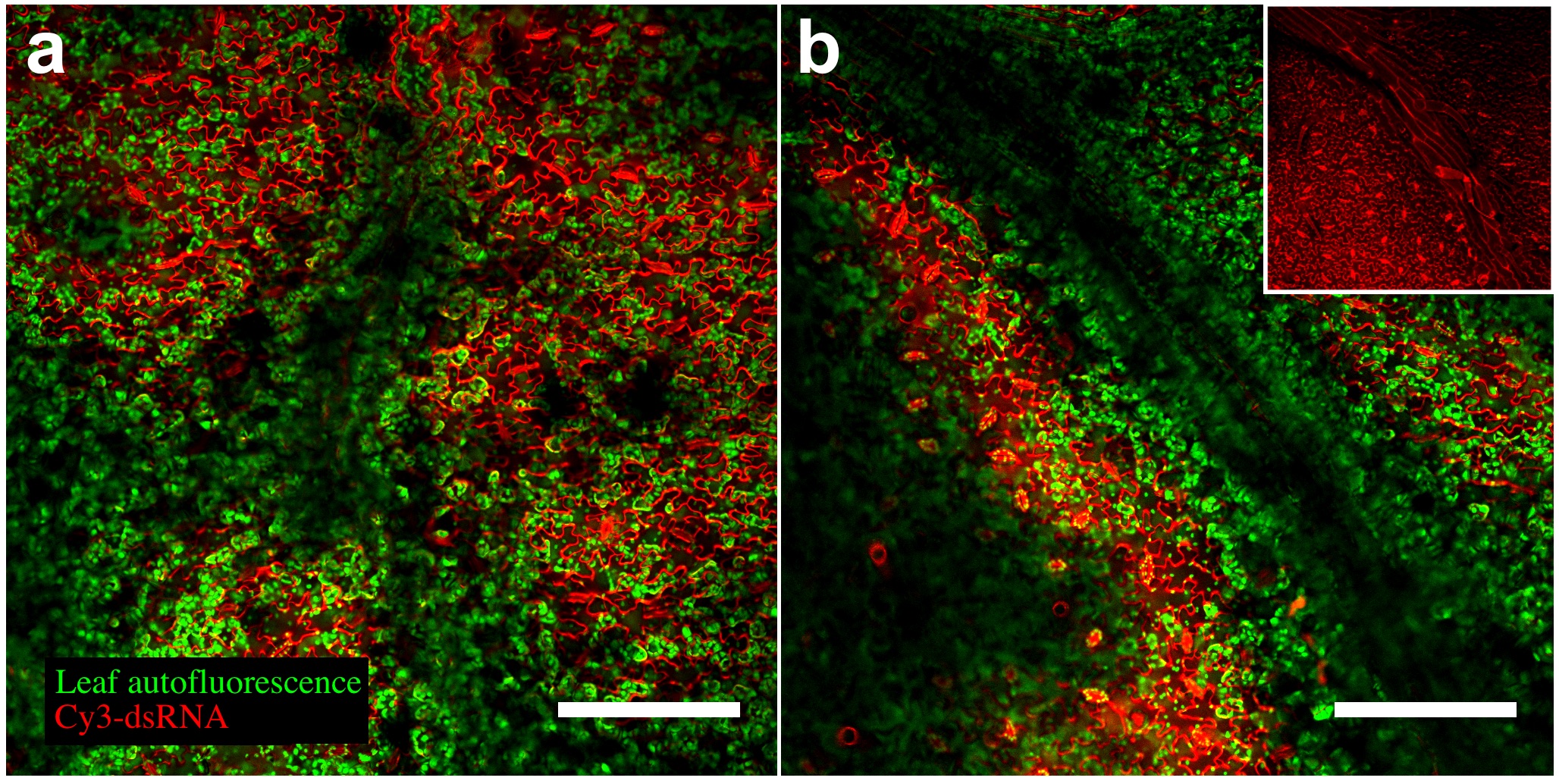

Supplement: Figure S2 — Detail of various regions on the treated leaf, indicating the dsRNA distribution over leaf areas (bar = 200 µm). [file peerj-04-2673-s002.jpg]

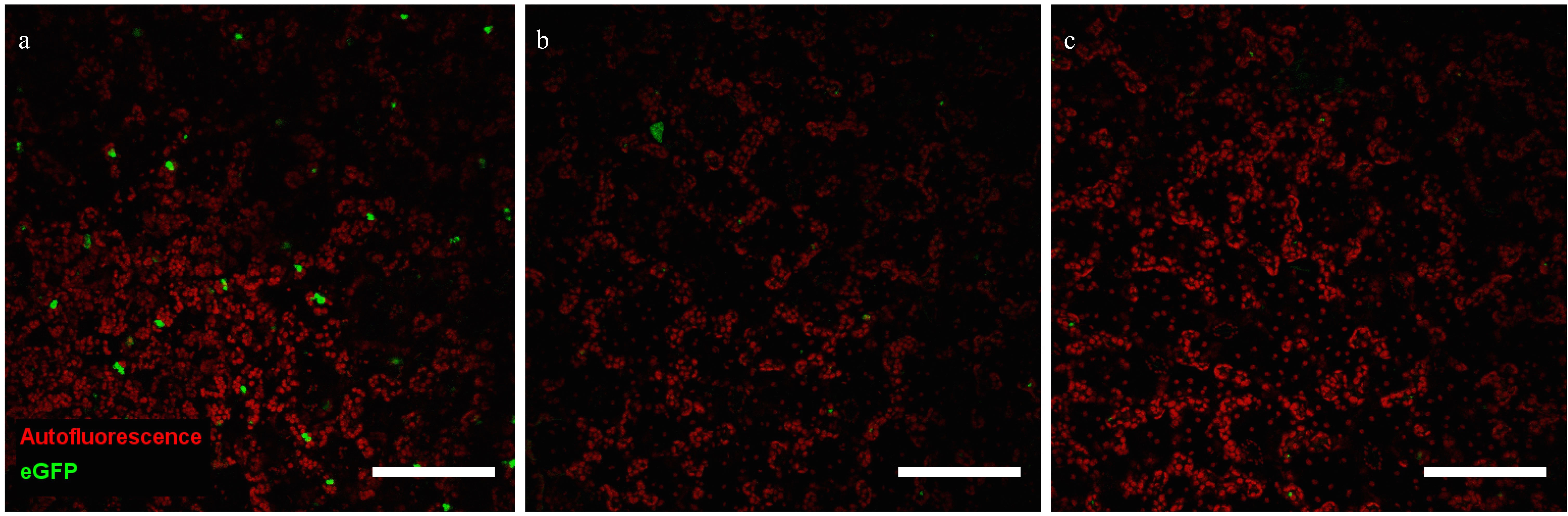

Supplement: Figure S3 — (A) Expression of eGFP in tomato leaf infiltrated with Agrobacterium suspension containing plasmid with eGFP, visualized under a confocal fluorescent microscope. (B) Infiltration of tomato leaf with two Agrobacterium clones, expressing eGFP together with a construct expressing GFPi, indicating reduction in GFP expression visualized under confocal fluorescent microscope. (C) tomato leaf tissues at a distance away from the area infiltrated with Agrobacterium suspension containing plasmid with eGFP (bar = 100 µm). [file peerj-04-2673-s003.jpg]

*GFP*

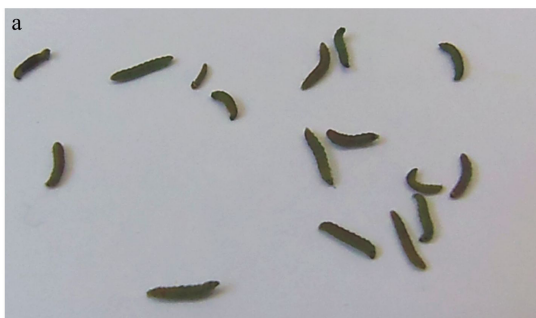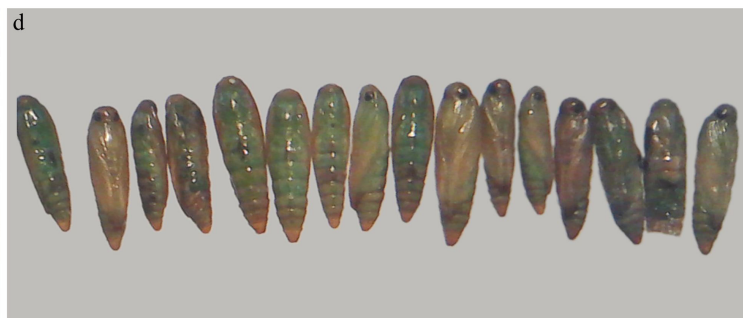

*V-ATPase*

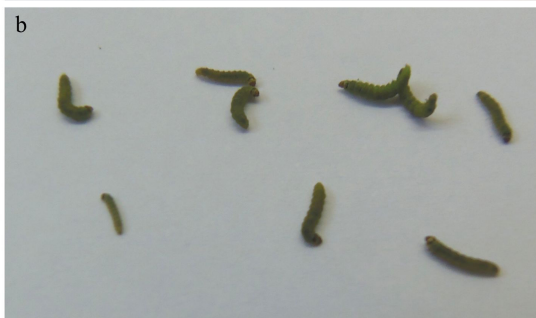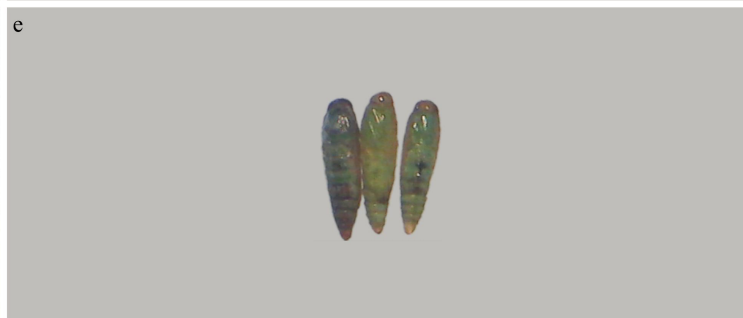

*AK*

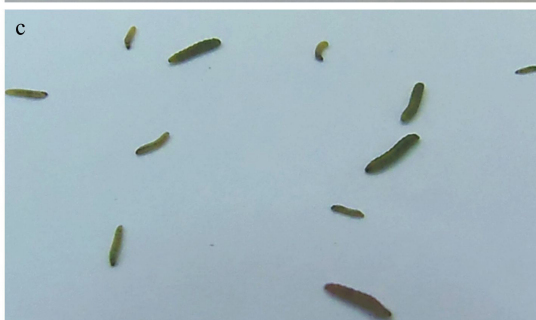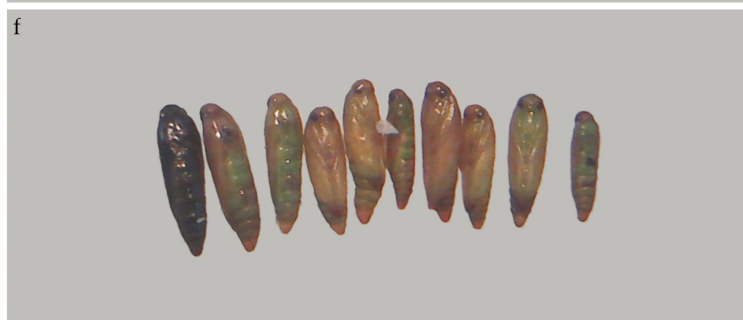

Supplement: Figure S4 — Tuta absoluta individuals after feeding for 11 days in tomato leaflets, after absorbing 500 ng dsRNA of the GFP control (a) and the target genes V-ATPase (b) or AK (c). Total amount of individuals at pupal stage, resultant from the larvae feeding on GFP control (d) and the target genes V-ATPase (e) or AK (f) [file peerj-04-2673-s004.pdf]

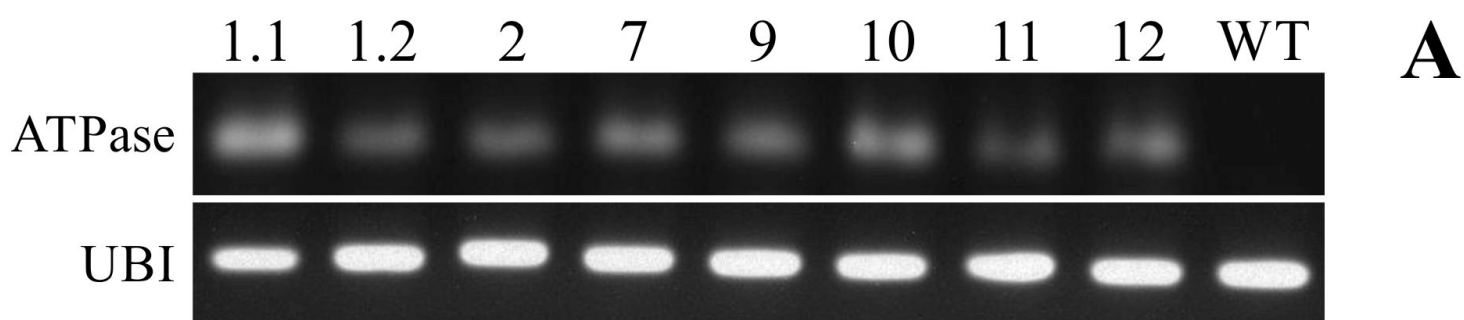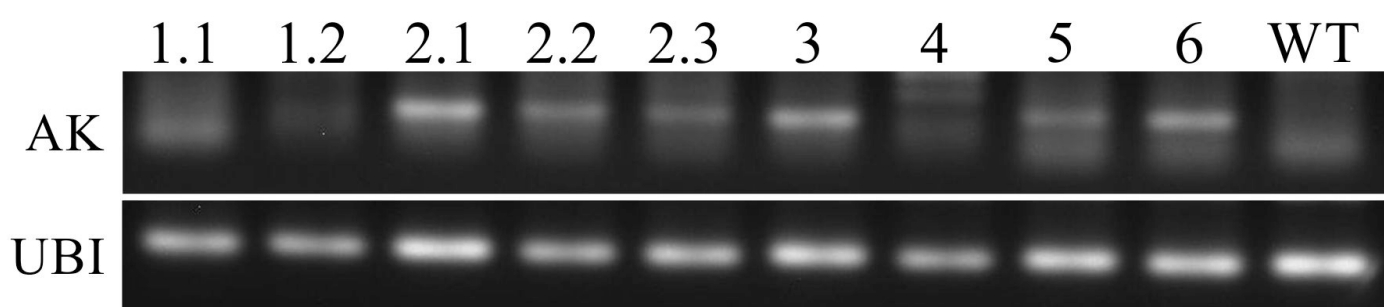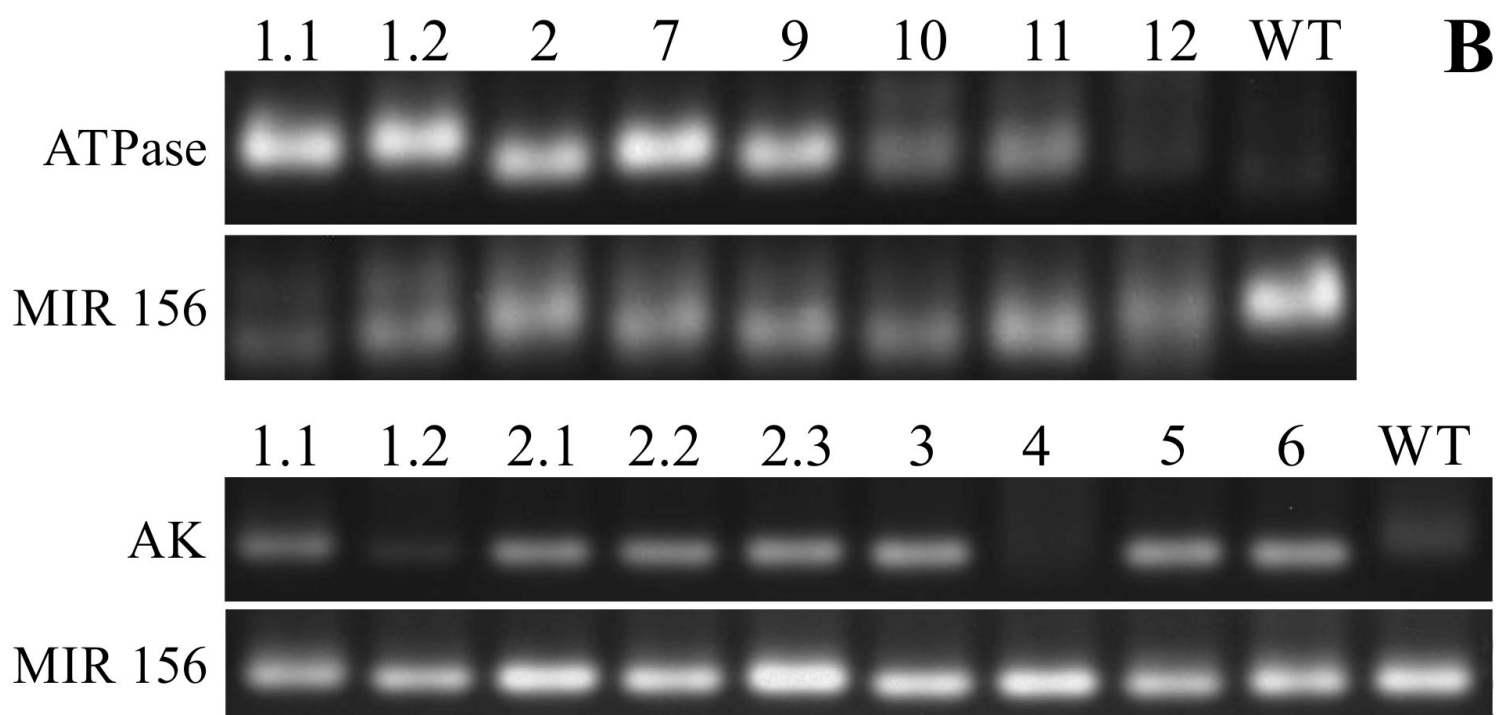

Supplement: Figure S5 — (A) Detection of amplification products from cDNA (RT-PCR) extracted from various transgenic ‘Micro-tom’ events and non-transformed control (WT) using primers specific for insect V-ATPase (top panel; 139 bp) or AK (bottom panel;190 bp), and tomato ubiquitin (108 bp). (B). Detection of amplification products derived from stem loop pulsed RT-PCR for potential siRNA derived from target genes (V-ATPase or AK, both 60 bp), plus the microRNA156 (MIR156; 60 bp) control ran at 3% agarose gel electrophoresis. Numbers represent events (1st number) or plants within events (2nd number). [file peerj-04-2673-s005.pdf]
