# Supplementary material for: RNA interference as a gene silencing tool to control Tuta absoluta in tomato (Solanum lycopersicum)
Source: PeerJ. 2016 Dec 15;4:e2673. doi: 10.7717/peerj.2673 (PMC5162399; doi:10.7717/peerj.2673)
Supplement: Table S1 — Degenerated primers used to amplify and clone respective candidate gene targets for RNAi, with expected amplicon size for each reaction. Numbers refer to primer order used in amplification reactions. [file peerj-04-2673-s006.pdf]

Table S1. Degenerated primers used to amplify and clone respective candidate gene targets for RNAi, with expected amplicon size for each reaction. Numbers refer to primer order used in amplification reactions.

Gene	Forward	Reverse	Expected size
Vacuolar ATPase subunit A	1. GTRGGNGTYATGGCNCANATHCA	1. TGNTCRAARTCNGCYTTDATYTT	1635 bp
(V-ATPase)	2. ATGGCNACNATHCARGTNTAYGA	2. TTNACNGGRTCYTTRCCYTTCAT	1590 bp
		3. ACNTGYTTRTARAANGGRCARAA	1431 bp
Arginine Kinase (AK)	1. GGNTTYAARAARACNGAYAARCA	1. CCRTCRTDCATYTCYTTNACNGC	762 bp
	2. GARDVNCARTAYAARGARATGGA	2. CCNCCYTCNGCYTCNGTRTGYTC	540 bp
		3. ARRTGRTCYTCYTCRTTRCACCA	261 bp

R = purine; Y = pyrimidine; M = A/C; K = G/T; H = A/C/T; B = C/G/T; V = A/C/G; N = all bases.
