# Supplementary material for: RNA interference as a gene silencing tool to control Tuta absoluta in tomato (Solanum lycopersicum)
Source: PeerJ. 2016 Dec 15;4:e2673. doi: 10.7717/peerj.2673 (PMC5162399; doi:10.7717/peerj.2673)
Supplement: Table S2 — Primers used to amplify target gene fragments with Gateway recombination borders attL1 and attL2 (underlined), with expected amplicon size (bp). [file peerj-04-2673-s007.pdf]

Table S2. Primers used to amplify target gene fragments with Gateway recombination borders *attL1* and *attL2* (underlined), with expected amplicon size (bp).

Gene	Sequence	Amplicon
ATPase	F: GGGGCCAACTTTGTACAAAAAAGCAGGCTCCGTATTGCGTACCGGCAAG<br>R: GGGGCCAACTTTGTACAAGAAAGCTGGGTACCTGTTTGTATAACGGGCAG	514 bp
Arginine Kinase	F: GGGGCCAACTTTGTACAAAAAAGCAGGCTGAGGCTCAATATAAGGAAATG<br>R: GGGGCCAACTTTGTACAAGAAAGCTGGGTTAAGATGGTCCTCTTCGTTGC	320 bp
