# Supplementary material for: RNA interference as a gene silencing tool to control Tuta absoluta in tomato (Solanum lycopersicum)
Source: PeerJ. 2016 Dec 15;4:e2673. doi: 10.7717/peerj.2673 (PMC5162399; doi:10.7717/peerj.2673)
Supplement: Table S3 [file peerj-04-2673-s008.pdf]

Table S3. Summary of three transformation experiments using construct containing repetitive and inverted gene fragments

	**No of explants**		**No. of regenerating plants**		**Transformation Efficiency**	
Experiments	*AK*	*V-ATPase*	*AK*	*V-ATPAse*	*AK*	*V-ATPase*
1	400	400	0	18	0.0%	4.5%
2	500	280	32	53	6.4%	18.9%
3	400	400	14	44	3.5%	11.0%
Total Regenerating Plants			46	115		
