# Supplementary material for: RNA interference as a gene silencing tool to control Tuta absoluta in tomato (Solanum lycopersicum)
Source: PeerJ. 2016 Dec 15;4:e2673. doi: 10.7717/peerj.2673 (PMC5162399; doi:10.7717/peerj.2673)
Supplement: Table S4 [file peerj-04-2673-s009.pdf]

Table S4. Specific primers designed for transcriptional analysis of gene-targets for silencing, with expected amplicon size in base pairs.

Gene	Primer Sequences		Amplicon (bp)
ATPase	F: ACCTGTCGGAGATCGTGCAG	R: AACGGGCAGAAACGGTCGTA	139
AK	F: GGCACATTCTACCCACTCAC	R: GATGGTCCTCTTCGTTGCAC	190
Rpl 5	F: CAGTCGTCGAGCCAGCAACA	R: TCCCGCATTGAAGGAGACCA	129
Rpl 23A	F: TTGACGCCATAACGTGGCAGT	R: CGCAAACGCCTGACTGTTCA	170
rRNA	F: TATGTTGTGAGGCGACGATG	R: GATCCACCGTCCAGGGTAAT	155
