# Supplementary material for: RNA interference as a gene silencing tool to control Tuta absoluta in tomato (Solanum lycopersicum)
Source: PeerJ. 2016 Dec 15;4:e2673. doi: 10.7717/peerj.2673 (PMC5162399; doi:10.7717/peerj.2673)
Supplement: Table S5 — Gene-specific and the universal primer sequences used to detect the predicted small interfering RNAs (siRNA) derived from the target genes V-ATPase (siRNA AATACATGCGCGCTCTAGATGAC) and AK (siRNA AAGTATCGTCCACACTGTCTGGC) and the control microRNA156 (UGACAGAAGAGAGUGAGCAC) in transgenic plants. [file peerj-04-2673-s010.pdf]

Table S5. Gene-specific and the universal primer sequences used to detect the predicted small interfering RNAs (siRNA) derived from the target genes *V-ATPase* (**siRNA** AATACATGCGCGCTCTAGATGAC) and *AK* (**siRNA** AAGTATCGTCCACACTGTCTGGC) and the control ***microRNA156*** (**UGACAGAAGAGAGUGAGCAC**) in transgenic plants.

siRNA/miRNA	Use	Primer
Vascular ATPase	RT	GTCGTATCCAGTGCAGGGTCCGAGGTATTCGCACTGGATACGACGTCATC
catalytic subunit A	PCR F	CCGGAGAATACATGCGCGCTC
Arginine Kinase	RT	GTCGTATCCAGTGCAGGGTCCGAGGTATTCGCACTGGATACGACGCCAGA
	PCR F	CGGCGGAAGTATCGTCCACAC
microRNA156	RT	GTCGTATCCAGTGCAGGGTCCGAGGTATTCGCACTGGATACGACTGCTCT
	PCR F	CCTGAGTGACAGAAGAGAGTG
	Universal R	GTGCAGGGTCCGAGGT
